# In‐Vivo Characterization of Blood‐Brain Barrier Permeability in Cognitively Normal APOE e4 Carriers and Non‐Carriers

**DOI:** 10.1002/alz.092054

**Published:** 2025-01-09

**Authors:** Seraphina K. Solders, Qian Shen, Charlotte S. Rivera, Sarah Banks, Emilie T. Reas

**Affiliations:** ^1^ University of California, San Diego, La Jolla, CA USA

## Abstract

**Background:**

An intact blood‐brain barrier (BBB) is critical for optimal brain health, and even transient or highly localized disruption of the BBB can have devastating consequences for neural functioning. BBB breakdown has been implicated in Alzheimer’s Disease (AD) pathogenesis, and elevated BBB permeability has been observed in APOE e4 carriers compared to non‐carriers. As prior studies have focused on a priori, AD‐vulnerable regions rather than whole‐brain analyses, little is understood about broader patterns of BBB breakdown that may relate to AD risk. This study aims to identify preclinical cerebrovascular differences in cognitively normal individuals who are genetically at high‐risk for AD.

**Method:**

Dynamic contrast‐enhanced magnetic resonance imaging (DCE‐MRI) was performed on 34 cognitively normal older adults (average age = 76.6 years; 18 women) from the UC San Diego Alzheimer’s Disease Research Center, evenly split by APOE e4 status. Data were processed using ROCKETSHIP and average BBB permeability values were extracted from the Desikan‐Killiany atlas cortical gray matter regions. Permeability values were averaged across hemispheres, resulting in 34 measurements. ANCOVAs predicted BBB permeability within each region from e4 status, age, sex, and the interaction between sex and e4 status.

**Result:**

APOE e4 carriers showed elevated BBB permeability throughout the superolateral frontal and parietal cortices and some occipital cortex compared to non‐carriers. The strongest effects were found in the caudal‐middle and superior frontal, pre‐ and paracentral, and superior parietal gyri. Further, there was a pattern of sex by e4 carrier status interaction whereby the e4 effect was stronger in men than women.

**Conclusion:**

Older adults who carry at least one APOE e4 allele show increased BBB permeability compared to e4 non‐carriers primarily in superior frontal and parietal regions, a spatial pattern that is notably distinct from the typical topography of AD pathology. This effect may be moderated by sex, such that men may be more vulnerable to the detrimental effects of APOE e4 on certain regions of the BBB compared to women. These findings raise implications for important questions regarding APOE4 dose effects, the mechanisms by which APOE regulates BBB functioning, and the evolution of BBB permeability through the disease course.

